# Automatic Extraction of Research Themes in Epidemiological Criminology From PubMed Abstracts From 1946 to 2020: Text Mining Study

**DOI:** 10.2196/49721

**Published:** 2023-09-22

**Authors:** George Karystianis, Paul Simpson, Wilson Lukmanjaya, Natasha Ginnivan, Goran Nenadic, Iain Buchan, Tony Butler

**Affiliations:** 1 School of Population Health University of New South Wales Sydney Australia; 2 School of Psychology University of New South Wales Sydney Australia; 3 School of Computer Science University of Manchester Manchestr United Kingdom; 4 Institute of Population Health University of Liverpool Liverpool United Kingdom

**Keywords:** epidemiology, study determinant, study outcome, PubMed, research priorities, epidemiological criminology, criminology, open research

## Abstract

**Background:**

The emerging field of epidemiological criminology studies the intersection between public health and justice systems. To increase the value of and reduce waste in research activities in this area, it is important to perform transparent research priority setting considering the needs of research beneficiaries and end users along with a systematic assessment of the existing research activities to address gaps and harness opportunities.

**Objective:**

In this study, we aimed to examine published research outputs in epidemiological criminology to assess gaps between published outputs and current research priorities identified by prison stakeholders.

**Methods:**

A rule-based method was applied to 23,904 PubMed epidemiological criminology abstracts to extract the study determinants and outcomes (ie, “themes”). These were mapped against the research priorities identified by Australian prison stakeholders to assess the differences from research outputs. The income level of the affiliation country of the first authors was also identified to compare the ranking of research priorities in countries categorized by income levels.

**Results:**

On an evaluation set of 100 abstracts, the identification of themes returned an *F*_1_-score of 90%, indicating reliable performance. More than 53.3% (11,927/22,361) of the articles had at least 1 extracted theme; the most common was substance use (1533/11,814, 12.97%), followed by HIV (1493/11,814, 12.64%). The infectious disease category (2949/11,814, 24.96%) was the most common research priority category, followed by mental health (2840/11,814, 24.04%) and alcohol and other drug use (2433/11,814, 20.59%). A comparison between the extracted themes and the stakeholder priorities showed an alignment for mental health, infectious diseases, and alcohol and other drug use. Although behavior- and juvenile-related themes were common, they did not feature as prison priorities. Most studies were conducted in high-income countries (10,083/11,814, 85.35%), while countries with the lowest income status focused half of their research on infectious diseases (47/91, 52%).

**Conclusions:**

The identification of research themes from PubMed epidemiological criminology research abstracts is possible through the application of a rule-based text mining method. The frequency of the investigated themes may reflect historical developments concerning disease prevalence, treatment advances, and the social understanding of illness and incarcerated populations. The differences between income status groups are likely to be explained by local health priorities and immediate health risks. Notable gaps between stakeholder research priorities and research outputs concerned themes that were more focused on social factors and systems and may reflect publication bias or self-publication selection, highlighting the need for further research on prison health services and the social determinants of health. Different jurisdictions, countries, and regions should undertake similar systematic and transparent research priority–setting processes.

## Introduction

### Background

Incarcerated populations experience poor health including chronic diseases, blood-borne viruses, sexually transmitted infections, and mental health problems [1]. Increased all-cause mortality has been described in those exposed to prisons, with the immediate postrelease period being a time of heightened susceptibility to suicide and drug overdose [2,3]. The health disparity between people in prison and the general population has been attributed to socioeconomic factors and health risk behaviors including tobacco smoking, alcohol consumption, and substance use [1,4,5].

To identify the health needs of incarcerated individuals, research is required to develop policies and programs that can address health inequities. The emerging field of epidemiological criminology studies the intersection between public health and justice systems, focusing on the prevalent health issues that affect offending and incarcerated populations. This area seeks to apply the scientific principles and methods of epidemiology and public health to criminal justice outcomes by framing crime and offending as a public health issue involving the inspection of health, well-being, and social and behavioral factors to explain and prevent patterns of offending [6,7].

Although investments in epidemiological criminology research have delivered some important benefits, health has received limited attention [8]. To increase value and reduce waste in research activity, transparent research priority setting is important and should consider the needs of research beneficiaries and end users along with a systematic assessment of existing research activities to assist in addressing gaps and harnessing opportunities [8]. Lund et al [9] proposed 4 essential elements to promote a systematic and transparent research priority–setting process: stakeholder involvement (ie, including research beneficiaries and end users), expert views, ranking methods, and a review of research published in peer-reviewed journals.

Correctional systems often publish research priorities for incarcerated populations, inviting proposals for research in these areas. However, the methods used to identify priorities, including whether stakeholders are consulted as part of the priority setting, are rarely disclosed. In addition, there is not enough published literature on stakeholders’ views on research priorities relating to the health of people in prison [10-14]. A recent study that attempted to determine the research priorities for incarcerated populations in Australia found that mental health was ranked as the top priority by prison health service directors and prisoners alike, with prisoners also ranking prison health services, alcohol and other drug use, education, and infectious diseases as key issues that need to be addressed [14]. Beyond this Australian perspective, such priorities are likely to resonate with prison systems in other parts of the world [15].

Following stakeholder consultation, a logical next step is to analyze the gap between research priorities and the existing research via publications. A source that can provide useful research activity insights into epidemiological criminology is PubMed, a bibliographical database developed by the National Library of Medicine, which covers millions of citations from biomedical journals [16]. Given the voluminous nature of published output, the task of reading and integrating relevant knowledge can be extremely challenging; therefore, any method to automatically extract such information from the relevant literature would be welcomed [17]. A limited number of studies have attempted to automatically extract information from epidemiological data [17-21]. de Bruijn et al [17] recognized key information (eg, eligibility criteria, sample size, and route of treatment) from randomized controlled trial (RCT) reports. The same approach was expanded by Kiritchenko et al [18] to extract additional characteristics (eg, primary outcome names and experimental treatments) from journal articles that reported RCTs. Other efforts included the recognition of phase III clinical trial information such as patient information [19] or the number of participants from RCTs [20] and the automatic identification of key characteristics (ie, study design, populations, exposure, outcome, confounding factors, effect size, and country where the study was conducted) from epidemiological study abstracts related to obesity and environmental health topics [21,22].

### Objectives

In this study, we applied a text mining method on 23,904 PubMed epidemiological criminology abstracts to extract the types of studied determinants and outcomes (hereafter referred to as “themes”). We mapped the extracted results (global and Australian results) against the identified research priorities from our previous study to assess the gaps between research activities and the research priorities of incarcerated people and prison health service directors. Leveraging from our previous work [23], we also identified the income level of the country of affiliation of the first authors to conduct a comparison between the ranking of research priorities and country groups by income levels.

## Methods

### Data Sample

Epidemiological criminology studies are indexed in bibliographical databases related to medicine such as PubMed. Thus, a literature search based on our original query [24] was conducted in PubMed using the query (given later in this section) to identify relevant studies [23]. We decided to use epidemiological abstracts, as they are written in a relatively structured format with their own reporting style that aims to standardize and improve study design and communication, making them ideal for the application of a rule-based text mining method [21].

To capture epidemiological studies, we used the Medical Subject Heading (MeSH) term “epidemiology” to ensure maximum specificity in the search. Because we used a variety of search terms to capture studies related to offenders and prisons, this prevented the inclusion of the articles that made only passing reference to prison work in the data set and resulted in a high-quality corpus for analysis. The search was restricted to articles published in English.

The full query (run on April 20, 2021) was as follows: “(*prison* or *borstal* or *jail* or *jails* or *engl* or *gaols* or *penitentiary* or *custody* or *custodial* or (*corrective* and (*service* or *services*)) or ((*correctional* or *detention*) AND (*centre* or *centres* or *center* or *centers* or *complex* or *complexes* or *facility* or *facilities*)) or (*closed* AND (*setting*)) or *prisoner* or *prisoners* or *incarcerated* or *criminals* or *criminal* or *felon* or *felons* or *remandee* or *remandees* or *delinquent* or *delinquents* or *detainee* or *detainees* or *convict* or *convicts* or *cellmate* or *cellmates* or *offenders* or *offender* or ((*young* or *adolescent*) AND (*offender* or *offenders*)) or ((*delinquent* or *incarcerated*) AND *youth*) or (*juvenile* AND (*delinquents* or *delinquent* or *delinquency* or *detainee* or *detainees* or *offender* or *offenders*)) or ((*young*) and (*people*) and (in) and (*custody*)) or ((*justice*) and (*involved*) and (*youth*)) or ((*incarcerated*) and (*young*) AND (*people* or *person* or *persons*)) or ((*juvenile* or *juveniles*) and (in) and (*custody*)) AND e*nglish*[lang] AND (“*epidemiology*”[Subheading] or “*epidemiology*”[MeSH Terms] OR *epidemiology*[Text Word]).”

### Theme Definition

In our study, we used the study determinants and outcomes in epidemiological study abstracts as “themes.” We considered a study determinant as a factor, characteristic, or other definable entity that brings about change in a health condition or in other defined characteristics, whereas outcome as the consequence of exposure in the population of interest, which in the case of epidemiological criminology includes offending and incarcerated populations. The themes ranged from mental and infectious illnesses (eg, schizophrenia and hepatitis C) to crime-related concepts (eg, recidivism).

### Dictionary

To identify the themes, we combined our rules with a manually crafted dictionary populated with terms of epidemiological criminology interest. We incorporated the most common infectious and mental health illnesses by including known synonyms, acronyms, and common abbreviations. Under the supervision of the third author (PS) and the last author (TB), additional and common law enforcement (eg, “jail sanctions,” “rearrests,” “arson,” and “sexual homicide”); social determinants (eg, “bullying” and “school connectedness”); and behavior-related concepts (“violence” and “aggressive behaviour”) were also included, resulting in roughly 2500 terms.

### Rule-Based Text Mining Approach

After inspecting a randomly selected sample of 100 abstracts (the training set), we based our rules on common syntactical patterns observed in the text that indicated the presence of a theme. The syntactical patterns made use of (1) frozen lexical expressions as anchors for certain elements built through specific verbs, noun phrases, and prepositions and (2) semantic placeholders, which were identifiable through the dictionary application that suggested a theme.

In the following example of a syntactical pattern (“*criminality* is a risk factor for *severe* *injury*”), to identify the themes of interest (“criminality, severe injury”), the semifrozen lexical expression “is a risk factor for” is matched via a regular expression containing variations of such terms (eg, risk factor, protective factor, and predictors), whereas “criminality” and “severe injury” obtain a match through the dictionary. More than 1 syntactical pattern may be matched in an abstract and may refer to ≥1 theme mention (that can be a duplicate).

General Architecture for Text Engineering [25] was selected for the design and implementation of the rules as well as for the annotation of the themes in the training and development sets. The observed syntactical patterns were converted into rules using the Java Annotations Pattern Engine, which is a pattern-matching language for General Architecture for Text Engineering. An additional (ie, development) set with 100 randomly selected abstracts was used to optimize the performance of the rules. Multimedia Appendix 1 shows some examples of the rules.

### Theme Standardization and Abstract-Level Unification

The extracted themes were manually inspected to standardize them. Abstracts which had themes with similar meanings (eg, an acronym or synonym) were manually assigned a respective term (eg, “HIV,” “AIDS,” and “human immunodeficiency virus” were assigned to “HIV”; “depression” and “depressive disorder” were assigned to “depression”). To obtain results at the abstract level for each study, we eliminated any duplicate mentions of the standardized themes.

### Mapping Themes to Stakeholder Research Priorities

We mapped the standardized themes to the research priority categories identified by stakeholder participants from our previous research to determine any alignment with the details of the methods and priorities reported elsewhere [13,14]. Themes unable to be mapped to any of the categories were assigned to 1 of the 5 new categories we created (“juveniles,” “offence related,” “bio and medical related,” “justice system,” and “behavior”). Any themes (eg, “age,” “accountability,” “church attendance”) that remained unmapped were assigned to an additional category named “other.”

This process was conducted by the first and the third authors (GK and PS), who manually mapped all the unique mentions of the standardized themes to their respective categories, with disagreements in mapping adjudicated by the last author (TB). The interannotator agreement observed was 94%, suggesting a reliable classification. Table 1 presents all categories including category focal points and examples of the mapped themes.

**Table 1:** Research priority categories identified by stakeholder groups and examples of mapped themes. The categories of hygiene and disability did not have any mapped themes.

Category	Stakeholder group	Category focal points	Examples of mapped themes
Mental health	Incarcerated people and prison health service directors	Serious mental illness, grief and trauma, medication access and management, access to psychologists, peer support, culturally appropriate care, and alternatives to isolation as a treatment option	Schizophrenia and psychosis
Health care service	Incarcerated people and prison health service directors	Access to and quality, delivery, human resources and financing, and outcomes of integrated prison health care services, including mental health care	Screening program and treatment completion
Alcohol and other drug use	Incarcerated people	Harm prevention, substance withdrawal, best practice for peer support, prison-based needle, and syringe programs	Nicotine addiction and cocaine abuse
Education (from health to vocational)	Incarcerated people	Development and evaluation of effective health education programs on drug withdrawal, treatments, and harm prevention; practicing good hygiene in and out of prison; and mental health self-care. Access to literacy programs, secondary school education, vocational training, and tertiary education	School performance and academic difficulties
Infectious disease and infections	Incarcerated people and prison health service directors	Hepatitis C, sexually transmissible infections, and tooth infections	HIV, HCV^a^, Rubella, and norovirus
Women’s and maternal health	Incarcerated people and prison health service directors	Nutrition, stress during pregnancy, specialized care and health plan for pregnant women, pregnancy education, staying away from children, isolation from family, and postnatal depression	Maternal connectedness and motherhood
Social determinants of health	Incarcerated people and prison health service directors	Poverty and inequality, education, discrimination and racism, employment and career, family attitudes, criminal justice, and system contact	Stable home environment and childhood sexual abuse
Disability	Incarcerated people	Mobility support and aids, and training programs for prisoners as carers	—^b^
Nutrition	Incarcerated people and prison health service directors	Better food nutrition in prison and specialized diets	Nutritional improvements and food supplements
Hygiene	Incarcerated people	Access to quality personal and accommodation cleaning supplies	—
Cognitive and intellectual disability	Prison health service directors	Cognitive and intellectual disability assessment and supports	Learning disability and intellectual disability
Postrelease health maintenance	Prison health service directors	Sustaining health gains made in prison in the community after release from custody	—
Aging prisoners	Prison health service directors	Aged care assessments	—
Workforce	Prison health service directors	Workforce development; ethical practice; and how to attract, train, and maintain the prison health workforce	—
Chronic health problems	Prison health service directors	Prevention and management of chronic health conditions in prison and in the community after release from custody	—
Aboriginal prisoner health	Prison health service directors	Health needs of Aboriginal and Torres Strait Islander prisoners	—
Complex needs	Prison health service directors	Assisting in better diagnoses of ≥2 conditions and assessment of needs affecting physical, mental, and social well-being	—
Justice system^c^	N/A^d^	Legal and procedural aspects of the justice system and its institutions	Sanctions, law and adjudication
Biomedical related^c^	N/A	Use of biology and physiology to understand, treat, and prevent disease	Injury, 5httplr, spinal cord injury, and age
Offense related^c^	N/A	Law enforcement, criminology, and offenses	Crime recidivism and homicide
Juveniles^c^	N/A	Juvenile delinquency	Adolescent delinquency and offspring disinhibited behavior
Behavior^c^	N/A	Human behavior	Acculturation, violence, male-male sex
Other^c^	N/A	Could not be mapped any of the above categories	Age, sex, gender, and job satisfaction

^a^HCV: hepatitis C virus.

^b^Not available.

^c^Categories including themes that could not be mapped to the research priority categories and were created by the authors.

^d^N/A: not applicable.

For each PubMed article, we used the country affiliation of the first author as described in previous research, with a total of 127 countries being identified [23]. Cases in which affiliations had >1 country were also included in the analysis. We classified countries into 4 categories (high income, upper-middle income, lower-middle income, and low income) based on the income status using the World Bank’s country classification schema [26].

### Ethical Considerations

No ethics approval was sought for this type of work as the data are all publicly available, do not have any identifying information and do not involve the authors recruiting human participants.

## Results

### Evaluation of the Text Mining Method

We evaluated the system’s performance at the abstract level by considering whether the themes (present as study determinants and outcomes) were correctly extracted from the text. We used the standard definitions of precision, recall, and the *F*_1_-score [27]. We defined *true positive* as the detection of either all the correct mentions of an exposure or outcome or the recognition of a number of mentions for 1 exposure or outcome, even if the system failed to pick up some mentions in an abstract. For example, if an exposure in 1 abstract is “hepatitis C” and there are 2 mentions in the text (“HPC” and “hepatitis C”), then the detection of either 1 or both mentions would be considered a true positive at the abstract level with “hepatitis C” as a theme in this abstract. This also applies in cases where >1 exposure or outcome is present. A *false positive* (FP) at the abstract level is the extraction of unrelated exposure or outcome mentions that were not annotated manually. A *false negative* (FN) is the unrecognized mentions of exposure or outcomes that were not extracted by the system. For example, if an abstract contains ≥1 mention of “hepatitis C” (either as an exposure or an outcome) and our method did not pick up any of these, then this theme would be classified as FN.

We randomly sampled 100 PubMed abstracts for the evaluation set, which were annotated for their theme by the first (GK) and the last (TB) authors. We calculated the interannotator agreement as the absolute agreement rate, with a value of 90% suggesting reliable annotations [28]. At the abstract level, the returned precision and recall were 92.1% and 88%, respectively, whereas the *F*_1_-score was 90%, indicating reliable performance. An observed (and expected) drop of approximately 3% (3.2%-3.8%) occurred from the training set to the evaluation set for all 3 metrics (Multimedia Appendix 2).

### Query Findings

**Figure 1:**
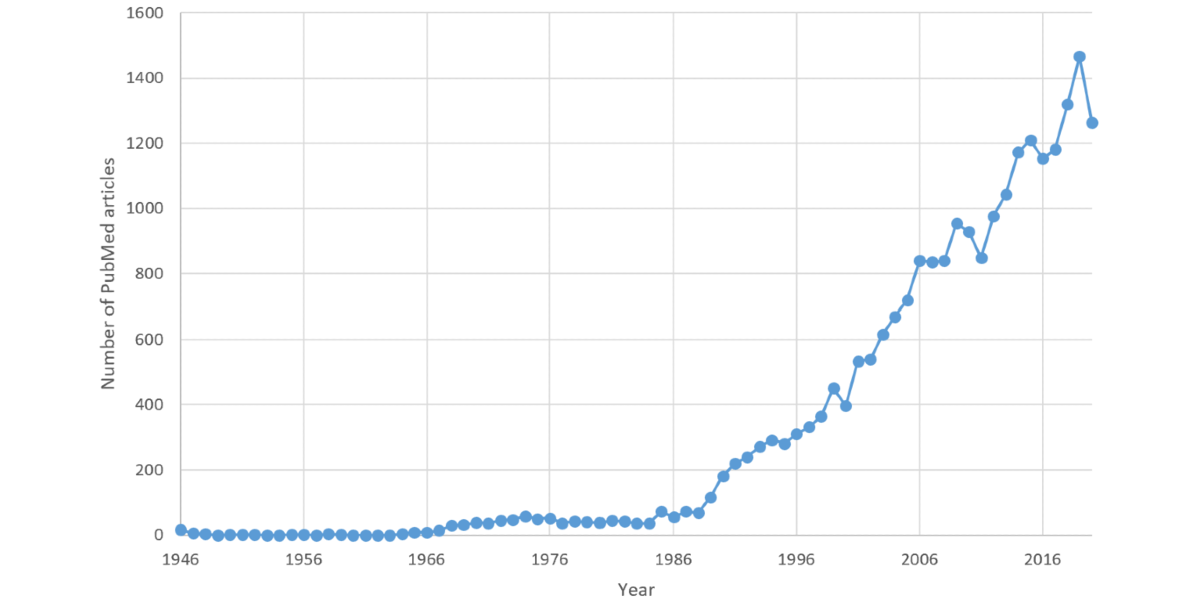
Number of published articles (n=23,722) in PubMed related to epidemiological criminology from 1946 to 2020. The results from 2021 (n=182) are not displayed.

The query returned 23,904 studies, with the earliest study recorded in 1946 (Figure 1). A total of 1543 (N=23,904, 6.45%) PubMed citations had no abstract, resulting in 22,361 (N=23,904, 93.6%) articles that were used in the text mining exercise. More than 50% (11,927/22,361, 53.3%) of the articles had at least 1 extracted theme, whereas overall, 600 distinct themes were identified. As the query was implemented in April 2021, results from that year were excluded (113/11,927, 0.95%), leaving 11,814 articles for the analysis. The reasons for this are outlined in the *Application to the Large Corpus* section.

**Figure 2:**
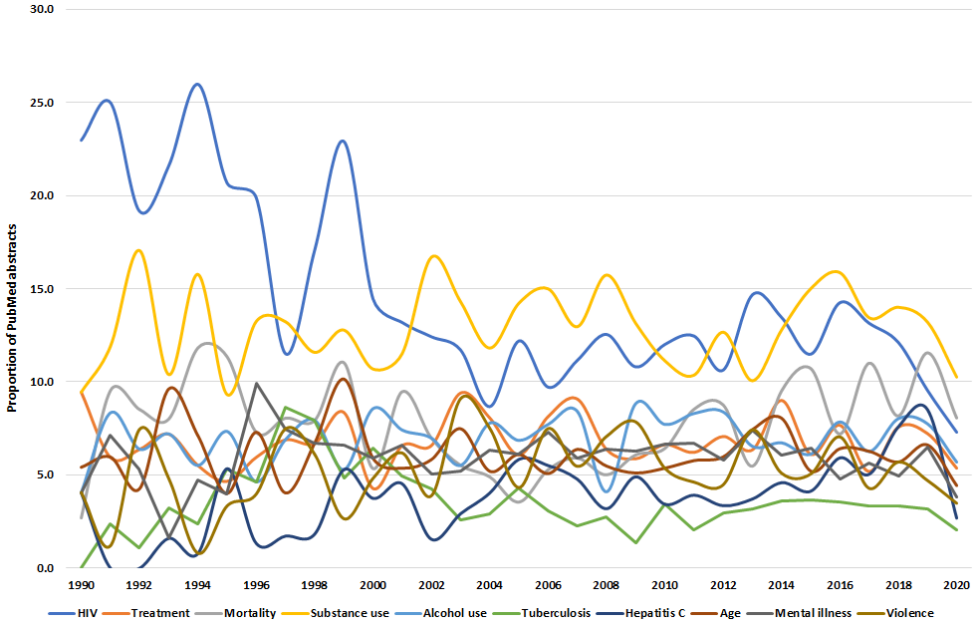
Proportion of epidemiological criminology PubMed articles (n=11,814) of the 10 most frequent themes annually from 1990 to 2020.

The most common theme was substance use (1533/11,814, 12.97%), followed by HIV (1493/11,814, 12.64%) and mortality (940/11,814, 7.96%; Table 2). If an abstract had >1 (ie, different) theme, these were counted separately. The “biomedical related” category had 6 of the most common themes within the top 20 (ie, mortality, age, sex, injury, gender, and youth). Although HIV was the most common theme, the proportion of these PubMed abstracts declined over the 30-year period (1990-2020; Figure 2). Although both alcohol and substance use showed stable abstract numbers, tuberculosis, after an initial peak in the mid-90s (ie, 1997), started to decline until 2020. By contrast, few articles proportionally examined hepatitis C in the 1990s, demonstrating an increase, particularly after and onward the mid-2000s (ie, 2005).

**Table 2:** The most frequent themes and their related stakeholder priority category in epidemiological PubMed abstracts from 1946 to 2020 (n=11,814).

Extracted variable	PubMed abstracts, n (%)	Mapping to stakeholder research priority category
Substance use	1533 (12.97)	Alcohol and other drug use
HIV	1493 (12.64)	Infectious disease and infections
Mortality	940 (7.96)	Biomedical related^a^
Alcohol use	837 (7.08)	Alcohol and other drug use
Treatment	818 (6.92)	Health care service
Age	721 (6.1)	Biomedical related^a^
Mental illness	704 (5.96)	Mental health
Violence	641 (5.42)	Behavior^a^
Hepatitis C	520 (4.4)	Infectious disease and infections
Tuberculosis	396 (3.35)	Infectious disease and infections
Crime	389 (3.29)	Offense related^a^
Injury	351 (2.97)	Biomedical related^a^
Offense	329 (2.78)	Offense related^a^
Depression	322 (2.72)	Mental health
Delinquency	314 (2.66)	Juveniles^a^
Sex	275 (2.32)	Biomedical related^a^
Recidivism	267 (2.26)	Offense related^a^
Gender	252 (2.13)	Biomedical related^a^
Youth	240 (2.03)	Biomedical related^a^
Hepatitis B	210 (1.78)	Infectious disease and infections

^a^Category created by the authors.

### Mapping Research Themes to Stakeholder Priorities

**Figure 3:**
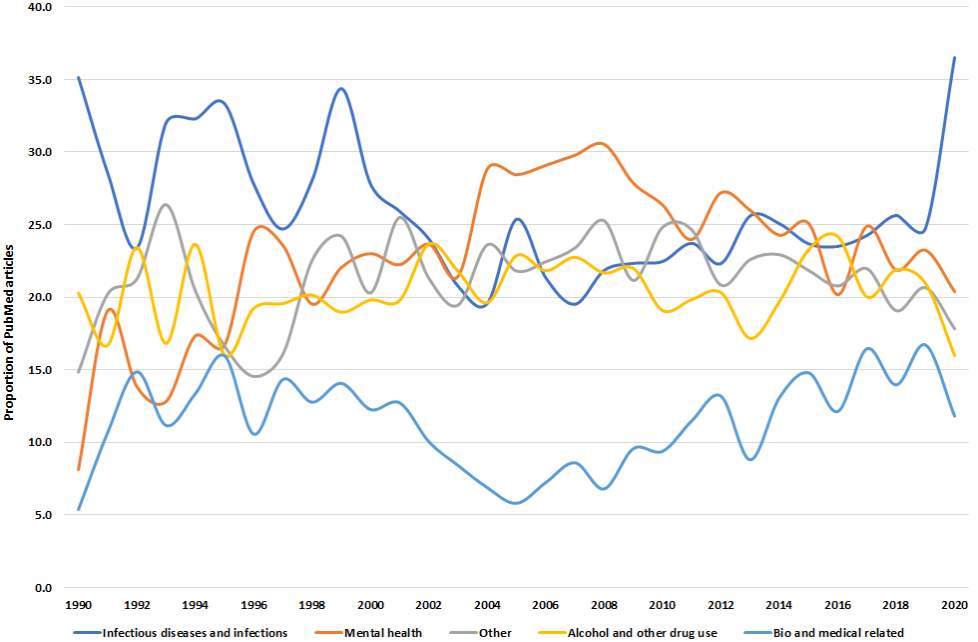
Proportion of epidemiological criminology PubMed articles (n=11,814) with the 5 most frequent research priority categories from 1990 to 2020.

In the 11,814 articles, infectious diseases and infections (n=2949, 24.96%) was the most common research priority category, followed by mental health (n=2840, 24.04%) and alcohol and other drug use (n=2433, 20.59%). Education appeared in <2% (172/11,814, 1.46%) of PubMed abstracts, whereas unclassified themes (ie, others) were the third most mentioned category (2552/11,814, 21.6%; Multimedia Appendix 3). Despite infectious diseases and infections having proportionally the highest percentage of PubMed articles in 1990 (26/74, 35%), this was followed by a gradual decline toward the mid-2000s and then a resurgence until 2020 (231/633, 36.4%). Although mental health had the second lowest proportion of articles (6/74, 8%) among the 5 most common categories in 1990, it increased after 2004 (100/347, 28.8%; Figure 3).

To explore whether it was reasonable to compare priority-setting categories determined by Australian stakeholders with all PubMed articles, we compared the 679 abstracts with Australia as the first author country affiliation to all PubMed abstracts (Multimedia Appendix 3, column 4). A similar pattern of research activity emerged between the entire PubMed article data set and Australian-identified abstracts. Given this similarity in the remaining analyses, we examined all the PubMed articles.

The most common themes (in terms of the number of PubMed articles) in the infectious diseases and infections category were HIV, hepatitis B, hepatitis C, tuberculosis, and COVID-19 (Table 3). Almost 50.63% (1493/2949) of the PubMed articles reported HIV as a research theme. For mental illness, specific mental disorders were researched, including depression at 11.34% (322/2840), followed by antisocial personality disorder at 6.83% (194/2840). In the alcohol and other drug use category, 34.4% (837/2433) of PubMed articles reported research on alcohol use and abuse, followed by 3.62% (88/2433) for cannabis use.

**Figure 4:**
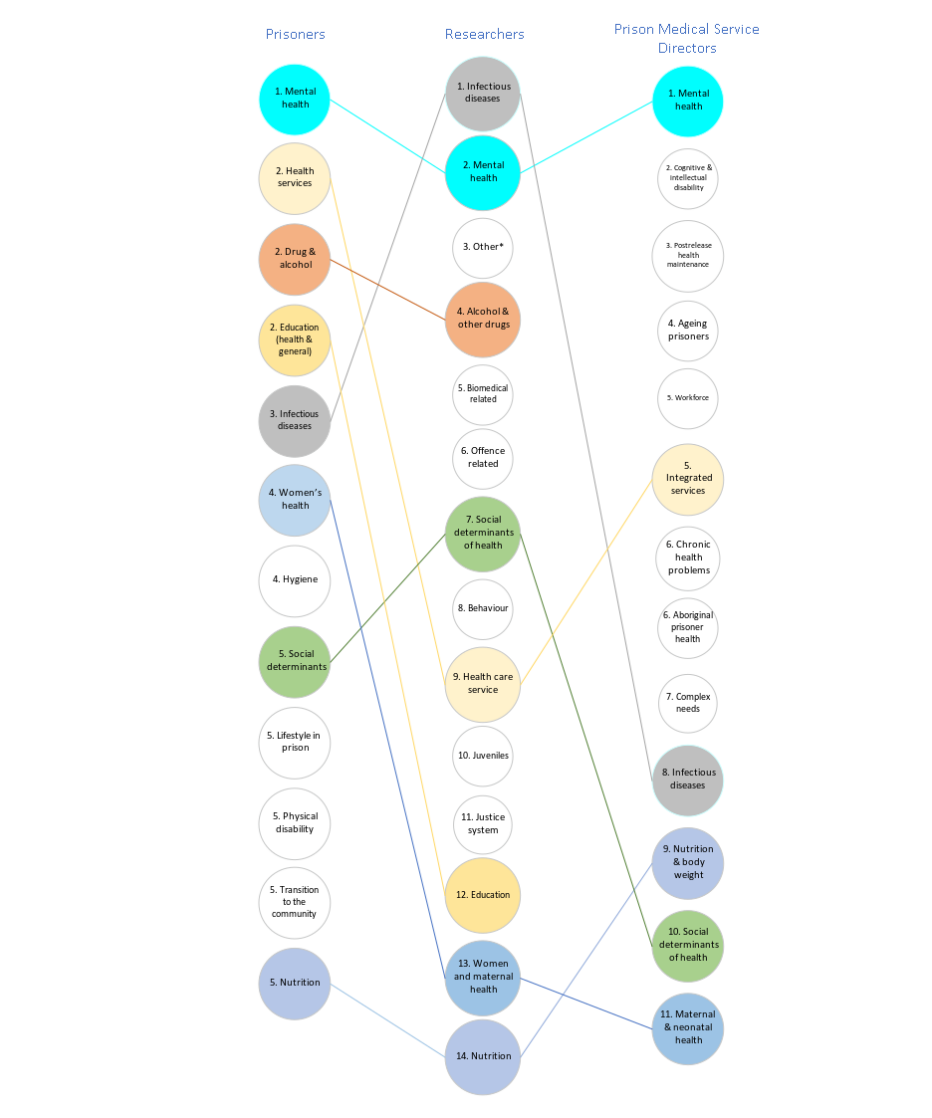
Mapping the identified researched themes from 11,814 PubMed epidemiological criminology abstracts from 1946 to 2020 to the research priority rankings of stakeholders (ie, prisoners and prison medical service directors). *Extracted themes that could not be mapped to any of the research priority categories identified by stakeholder groups.

Comparing the text-mined (then mapped) research themes with the stakeholder research priorities revealed an alignment across the 3 groups (ie, prisoners, researchers, and prison medical service directors) for mental health, infectious diseases, and alcohol and other drug use, as well as some notable differences or gaps (Figure 4). Both incarcerated participants and prison health service directors identified cognitive and intellectual disabilities and transition to community or postrelease health maintenance as research priorities; however, these were not included in the PubMed research activities. Although incarcerated people identified hygiene, nutrition, and lifestyle in prison as priorities, there has been little PubMed research activity in these categories. Research activities on health care services, education, and women or maternal health were low compared with the priority rank; these categories were given by incarcerated participants (and prison health service directors for integrated services).

**Table 3:** Frequency of extracted themes from PubMed abstracts (n=11,814) for the 3 most common research priority categories (excluding “Other”) from 1946 to 2020 (n=11,814).

Categories			PubMed abstracts^a^, n (%)
**Mental health (n=2840)**			
	Mental illness	704 (24.79)	
	Depression	322 (11.34)	
	Antisocial personality disorder	194 (6.83)	
	Posttraumatic stress disorder	178 (6.27)	
	Attention-deficit/hyperactivity disorder	135 (4.75)	
	Psychopathology	120 (4.22)	
	Psychopathy	117 (4.12)	
	Depressive symptom	110 (3.87)	
	Anxiety	109 (3.83)	
	Schizophrenia	97 (3.41)	
**Infectious diseases and infections (n=2949)**			
	HIV	1493 (50.63)	
	Hepatitis C	520 (17.63)	
	Tuberculosis	396 (13.43)	
	Hepatitis B	210 (7.12)	
	Sexually transmissible infection	113 (3.83)	
	COVID-19	108 (3.66)	
	Chlamydia	93 (3.15)	
	Sexually transmissible disease	85 (2.88)	
	Malaria	66 (2.24)	
	Hepatitis	65 (2.2)	
**Alcohol and other drug use (n=2433)**			
	Substance use	1533 (63)	
	Alcohol	837 (34.4)	
	Cannabis	88 (3.62)	
	Cocaine	41 (1.69)	
	Adolescent substance use	30 (1.23)	
	Intravenous drug	24 (0.98)	
	Opioids	14 (0.57)	
	Nicotine	13 (0.53)	
	Alcohol-related harm	11 (0.45)	
	Heroin	11 (0.45)	

^a^PubMed abstracts with >1 extracted theme were counted separately.

### Outputs From Low-, Lower-Middle–, Upper-Middle–, and High-Income Countries

Most studies derived from PubMed (N=11,814) were from high-income countries (n=10,083, 85.35%), whereas low-income countries accounted for >1% (n=91, 0.77%; Multimedia Appendix 4). Upper-middle–income and lower-middle–income countries had 6.26% (740/11,814) and 3.68% (435/11,814) of PubMed articles, respectively. Furthermore, 4.14% (489/11,814) articles did not have any affiliated country.

**Figure 5:**
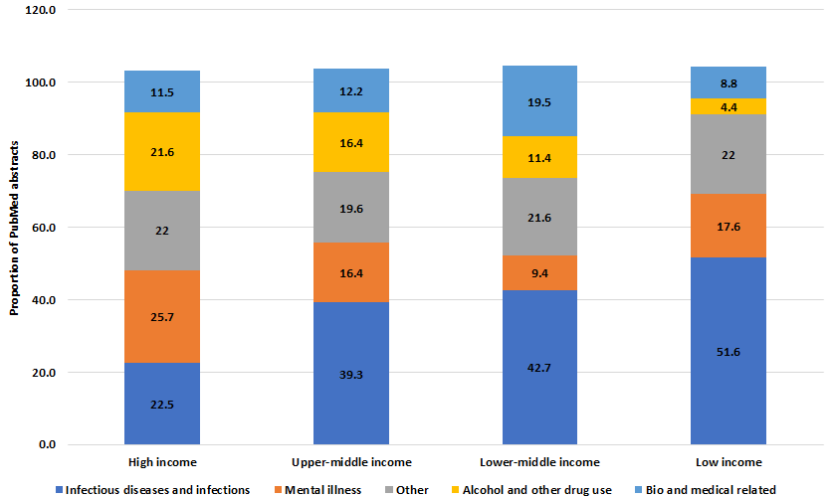
Most frequent research priority categories by classification of country income for the period 1946 to 2020 from 11,814 PubMed epidemiological criminology abstracts.

High-income countries focused one-fourth of their research on mental health (2590/10,083, 25.69%) as opposed to 9.4% (41/435) in lower-middle–income countries and 18% (16/91) in low-income countries. Those with lower-income status focused their research on infectious diseases, particularly the lower- and low-income countries, with 42.7% (186/435) and 52% (47/91), respectively (Figure 5). Furthermore, 21.64% (2182/10,083) of research from high-income countries was on alcohol and other drug use, almost 5 times more than those from low-income countries (4/91, 4%; Figure 5).

For research themes that were not among the top 5, it should be noted that education and women’s and reproductive health were ranked at the bottom in all country income groups (0.1%-2.5%, Multimedia Appendix 4). Notable is the proportion of research on health care services in low-income countries (13/91, 14%), which is more than double that of high-income countries (794/10,083, 7.87%) and almost 3 times higher than that of upper-middle–income (41/740, 5.5%) and lower-middle–income countries (29/435, 5.9%; Multimedia Appendix 4).

## Discussion

### Principal Findings

This study assessed 70 years of epidemiological criminology research by automatically extracting research themes from 22,361 PubMed abstracts through the application of a rule-based method and mapping them against identified research priorities identified by Australian prisons and prison medical service directors [14]; we also examined the alignment of these priorities with published research globally, including a breakdown by income group of the countries involved.

Overall, of the 11,814 published articles that had an extracted research theme, there was a large decade-by-decade increase in research activities from 1986 onward. A number of plausible factors may have contributed to this exponential rise in research activities toward the health of incarcerated populations. For example, the “tough on crime” and “the war on drugs” approaches started in 1971 in the United States and led to increasing imprisonment rates and prison overcrowding [29], HIV emerged in the 1980s, and key 1970s events such as the riots in Bathurst Prison (Australia) prompted the creation of the Nagle Royal Commission, which exposed a culture of poor management and abuse in 2 New South Wales prisons, and the Royal Commission Into Aboriginal Deaths in Custody (1987-1990) [30-32]. The occurrence of such events could be considered a catalyst for the increased attention to prison conditions and the health of those in prison and potentially explain the post-1980 increase in published research on prisoner health.

The research priorities of our stakeholder groups in terms of the frequency of research output were infectious diseases or infections, mental health, and alcohol and other drug use. Some differences in the frequency of research activities undertaken were noted in these categories according to a country’s income level and between all studies published and studies relevant to only Australia. High-income countries focused one-fourth (25.7%) of their research on mental health, in contrast to 8.4% in lower-middle–income countries and 17.6% in low-income countries. Just over one-fifth (21.6%) of the published articles from high-income countries were devoted to alcohol and other drug use, nearly 5 times more than those in low-income countries. Lower-income countries focused most of their research on infectious diseases (38%-51.6%), compared with 22.5% in high-income countries. These differences based on a country’s income level are likely to be explained by local health priorities that possibly reflect an emphasis on easily treated conditions that pose a more immediate risk to life, whereby infectious diseases such as HIV and tuberculosis take policy precedence over mental health and alcohol and substance use disorders in lower-income countries. More attention and investment are directed toward mental health conditions and alcohol and substance use disorders in high-income countries. These different priorities may also help explain the minor differences found between all PubMed published articles and only those relevant to Australia regarding the proportionate differences between mental health, infectious diseases, and alcohol and other drug use.

Looking at each category, most infectious disease research activities concerned HIV. Although the number of articles on HIV began to decrease 2016 onward, an increase in studies on hepatitis C resulted in a rise in the overall number of infectious disease articles in PubMed. These trends likely reflect historical developments in disease prevalence and advances in treatment. In the case of hepatitis C, direct-acting antiviral medication became available in 2013, which offered significant improvements in efficacy, reduced side effects, and a shorter treatment duration [33]. Interestingly, COVID-19, despite the first known case emerging in December 2019, featured as a research theme in 3.66% (108/2949) of infectious disease category articles, higher than sexual transmissible disease (85/2949, 2.88%) and malaria (66/2949, 2.24%), indicating the concern posed by this new viral illness within the correctional system space.

Although the broad term “mental illness” featured as a research theme in most mental health category articles, depression, antisocial personality disorder, and posttraumatic stress disorder were the most frequent theme disorders extracted. In the alcohol and other drug use categories, almost one-third of the articles were related to alcohol (837/2433, 34.4%), followed by cannabis (88/2433, 3.62%). Given the public and media interest in amphetamine use and narratives connecting crime with amphetamine use, it is surprising that research activities did not necessarily reflect this topic [34].

Notable gaps were identified between the stakeholder research priority areas and research activity outputs. Prison health services and education (health and vocational) were the second most often ranked priorities by the incarcerated populations and endorsed by the World Health Organization as an important policy and research area [15]. However, as research theme categories, they received 7.71% (911/11,814) and 1.46% (172/11,814) of PubMed articles, respectively. In addition, only 9.71% (1147/11,814) of PubMed articles reported a theme within the social determinants of the health research priority category. This may be an artifact of publication bias or self-publication selection, that is, these stakeholder research priorities are more focused on social factors and systems, and thus, authors may tend to prefer publishing in less biomedically oriented journals that feature in other bibliographic databases. Nonetheless, given the developments in and the growing recognition of health services research [35] and the importance that quality health services are likely to have for incarcerated people, we contend that more research should be conducted and published in this area.

To examine whether the research priorities were current, we also mapped the themes from the last 10 years only (ie, 2010-2020) and observed them to be almost identical in rank to those from 1946 to 2020.

The stakeholder research priorities of women’s and maternal health featured in <0.11% (13/11,814) of the PubMed articles. This may reflect that historically, women have been underrepresented in incarcerated populations despite having drastically increased incarceration rates in recent years [36]. However, the reporting style of some epidemiological criminology studies might omit details of the gender aspect of certain research themes in the abstract, focusing instead on the general research aspects of incarcerated populations. This argument could also be extended to lower-priority research areas (see the *Application to the Large Corpus* section). A lack of interest in research on nutrition in prison may stem from researchers assessing the issue as a less pressing matter when compared with infectious disease transmission, posttraumatic stress disorder, or opiate substitution therapy. Given the encroaching privatization of correctional systems and administrative pursuits to provide system efficiencies and cost savings, nutrition services are likely to be the easy targets for efficiency savings [37] and thus likely warrant further research investigation.

Finally, disability, both physical and intellectual, despite being stakeholder research priorities, had little research activity. In the research priority category of mental health, learning disability had only 1.19% (34/2840) of articles. Although incarceration of people with disabilities is not a new phenomenon, this population and relevant support have been poorly understood even though incarceration rates of people living with a disability saw an increase in recent years [38], indicating that more research should be conducted to understand the needs of this susceptible population.

### Text Mining Error Analysis

#### Overview

A rule-based method was used that allowed automatic extraction of the studied themes (ie, study determinants and outcomes) from PubMed abstracts. The evaluation of the method demonstrated good performance with an overall 90% *F*_1_-score, suggesting that a rule-based approach can generate reliable results in epidemiological criminology despite the volatile nature of the targeted themes that range from mental illness to behavior-related concepts.

Our method combined a large manually crafted dictionary and rules based on common syntactical patterns that indicate the presence of an outcome and a study determinant to identify the themes investigated in epidemiological criminology studies. Despite the small number of rules (61), which were based on 2 relatively small (training and development) data sets, their scope proved useful for capturing variables of interest without the generation of large numbers of FPs or FNs. However, recognizing these themes (irrelevant of their role within the study context as study determinants or exposures or outcomes) came with some challenges.

#### Causes of FPs

Our approach identified only a part of the study determinants or outcomes (eg, “*problems* [FP] *in their social integration*”) because the way they were described was far from just a simple term that was present in our dictionary. Another source of FPs was the recognition of unrelated study concepts that were wrongly recognized as investigated themes due to the generic nature of our rules. In the following example, “violent offenders in State prisons are increasingly likely to report having used *alcohol* [FP] before committing their offenses, possibly illustrating the effect of more severe sanctions for alcohol-involved offenses,” “alcohol” was extracted although the abstract was focused on the effect of “more severe sanctions for alcohol-involved offences.” This suggests that a sentence-focused rule-based method may struggle to identify the main study determinant; thus, a wider context might need to be considered.

#### Causes of FNs

Because our dictionaries were limited by their manual design and included a more mental health–, infectious disease–, and crime-related focus, concepts that were not necessarily classified as such (eg, “staff perceptions,” “autonomic arousal,” and “dental caries experience”) or that had mentions that extended beyond single-word concepts (eg, “hearing, auditory processing, and language skills” and “law restricting access to firearms by domestic violence offenders”) were not identified (FNs). The incorporation of extensive sociology- and criminology-related terms into the dictionary might allow for greater coverage and potentially enhance the extraction of such themes.

In addition, certain variables based on generic patterns (eg, “*Psychiatric symptoms* [FN] among juveniles incarcerated in adult prison” and “*Deaths* [FN] in juvenile justice residential facilities”) were not identified because of a lack of such rules. Their implementation in our approach might lead to an increase in the number of FPs. Finally, some themes were not identified, as their syntactical patterns (eg, “*behavioral regulation dysfunction* [FN] was predictive on...” and “*ECF* [FN] was more strongly related to aggression”) had not been encountered in our training and development sets and hence were not incorporated in the current rule design.

### Application to the Large Corpus

Despite good results in both the development and evaluation sets (ie, *F*_1_-score of 92.7% and 90%, respectively), the application of this method in the 22,361 epidemiological criminology abstracts revealed that only in slightly >50% (n=11,927, 53.3%) of the articles, we extracted research themes. Because we included a more diverse literature space from epidemiological criminology and considered all study design types that could range from the relatively strict reporting structure of a clinical trial to the informal style that certain observational designs bear, it is not surprising to note that a certain number of articles did not explicitly state their research theme, at least in the abstract text.

To further test the robustness of our method, a random sample of 50 articles was inspected by the first and the last authors (GK and TB) to examine whether any studied variables were mentioned in the abstract text. We found FNs (12%) in 6 abstracts due to the lack of related terms from our dictionary (eg, “female problem gambling” and “assessment of prison life”) and the lack of implemented rules that was based on extremely common generic patterns, both explained as sources of FNs in the *Text Mining Error Analysis* section. Other reasons that might have contributed to the low number of abstracts with extracted themes were as follows: articles in the sample that were not epidemiological studies (eg, reports, technical notes, and opinion pieces; 23/50, 46%); articles that were epidemiological studies but not conducted in the area of criminology (16/50, 32%); 1 (N=50, 2%) abstract that was animal based; and 4 (N=50, 8%) abstracts did not state their research variable (eg, “characteristics that preceded a crime”). This analysis indicates that even though our system might miss some abstracts’ research themes, its results are reliable enough to indicate a map regarding the research priorities of marginalized populations who come in contact with the justice system.

### Limitations

Our study has several limitations. First, PubMed articles might not be sufficient to capture an accurate picture of offending and incarcerated populations, as relevant government articles and reports are often not published in academic journals. Furthermore, studies with a more sociological and criminal focus are unlikely to appear in journals covered by PubMed. Therefore, our data set likely underestimated the total number of research outputs in this area. In addition, our query may not be broad enough to capture all related articles in this area due to the use of a MeSH term (ie, “epidemiology”). The inclusion of extra MeSH terms such as “clinical trial” and “observational study” could potentially increase the number of articles that could provide a different picture in theory. The study also carries the risk of “English-language bias,” as including non-English articles presented resource challenges in terms of prospective costs, time, and expertise in non-English languages. The inclusion of non-English articles would help ensure greater generalizability and reduce bias [39].

The focus on abstracts might indicate a problem of theme representation, as we did not use full-text articles because it is possible that explicit information about study determinants and outcomes might not be available. It would be interesting to explore whether applying this method to full-text articles would improve the extraction performance. In addition, the type of the written abstract might have contributed to the generation of FPs and FNs. Although we designed the rules based on common lexical patterns that suggest the presence of a research theme, less structured abstracts might make it difficult to recognize such information automatically because structured abstracts offer higher-quality information as opposed to those that rely on free text [40].

Although the performance of our rule-based approach is reliable, the number of identified themes could be underrepresented. Using the manually crafted dictionaries based on terms, as opposed to more nuanced and complex concepts of cross-disciplinary nature that are described in sentences, could have resulted in a number of FNs, so the dictionary coverage could be limited. Although a relatively small number of rules were used to identify the study determinants and outcomes in the text, it is possible to have a restrained scope as it was shown with FNs that were not extracted due to a lack of implementation within the rule set. The application of other methods (eg, topic modeling) could potentially decrease the number of FNs.

### Conclusions

The identification of research themes (as study determinants and outcomes) in a large-scale sample of epidemiological criminology PubMed abstracts is feasible by using a rule-based approach with reliable performance (*F*_1_-score=90%). Recognizing themes can be useful in highlighting research gaps and potential biases in epidemiological criminology. We observed that the highest research activity concerned infectious diseases, mental health, and alcohol and drug use, which were the 3 research areas that were identified as priorities in our previous study by people in Australian prisons and Australian prison health service directors. The gaps between these stakeholder research priorities and actual research activity demonstrate the potential need for further research on prison health services, social determinants of health, women’s and maternal health, nutrition, and disabilities. We recommend others in different jurisdictions, countries, and regions to undertake similar systematic and transparent research priority–setting processes that include stakeholder involvement, expert views, and reviews of conducted research [9]. Such processes can be considered in other disciplines and fields.

## Appendix

Multimedia Appendix 1 Examples of rules for identifying themes from PubMed abstracts.

Multimedia Appendix 2 Precision, recall, and F1-score results for the training (n=100), development (n=100), and evaluation (n=100) sets, including the number of true positives, false positives, and false negatives at the document level.

Multimedia Appendix 3 The most common research priorities in 11,814 PubMed epidemiological criminology articles from 1946 to 2020.

Multimedia Appendix 4 Distribution of PubMed articles (n=11,814) according to the categorized theme and income status of the country of research origin for the period from 1946 to 2020.

## Abbreviations

FN: false negative
FP: false positive
MeSH: Medical Subject Heading
RCT: randomized controlled trial

## Data Availability

The data sets used in this study are publicly available in PubMed by implementing our query described in the *Data Sample* subsection under the *Methods* section.
