# Supplementary material for: Automatic Extraction of Research Themes in Epidemiological Criminology From PubMed Abstracts From 1946 to 2020: Text Mining Study
Source: JMIR Form Res. 2023 Sep 22;7:e49721. doi: 10.2196/49721 (PMC10559193; doi:10.2196/49721)
Supplement: Multimedia Appendix 1 [file formative_v7i1e49721_app1.docx]

**Multimedia Appendix 1**

[Examples of rules for identifying themes from PubMed abstracts.](https://formative.jmir.org/api/download?filename=c5650c9438db7fd7ca856c634ece0461.docx&alt_name=49721-776439-1-SP.docx)

| **Rule** | {Token.string==~"(?i)their\|its"} | {Token.string==~"(?i)associations\|association\|relationships\|relationship\|relation\|relations\|link\|links\|correlation\|interrelation\|connections\|connection\|interrelations\|correlations"} | | {Token.string==~"(?i)to\|with"} | (variables) |
| --- | --- | --- | --- | --- | --- |
| **Example** | its | relation | | with | HIV |
|  | | | | | |
| **Rule** | (variables) | {Token.string==~"(?i)were\|are\|is\|was"} | {Token.string==~"(?i)constructed\|examined\|inspected\|investigated\|explored\|assembled"} | {Token.string==~"(?i)to"} | {Token.string==~"(?i)address\|investigate\|explore\|inspect\|examine\|determine"} |
| **Example** | HIV | was | examined | to | address |

The rules use lenient token matching (lowercase or uppercase). {Token.string==∼”(? i)their|its”} will match any of the possessive nouns “their” or “its”; {Token.string==∼”(? i)to|with”} will match the preposition “to” or “with”; {Token.string==~"(?i)were|are|is|was"} will match any of the following: “were”, “are”, “is”, and “was”; same principle applies for any part of the rule that has “Token.string”; (variables) is a dictionary that contains several epidemiological criminology terms of interest including known abbreviations, acronyms and synonyms.
