# Supplementary material for: Automatic Extraction of Research Themes in Epidemiological Criminology From PubMed Abstracts From 1946 to 2020: Text Mining Study
Source: JMIR Form Res. 2023 Sep 22;7:e49721. doi: 10.2196/49721 (PMC10559193; doi:10.2196/49721)
Supplement: Multimedia Appendix 2 [file formative_v7i1e49721_app2.docx]

**Multimedia Appendix 2**

[Precision, recall, and F1-score results for the training (n=100), development (n=100), and evaluation (n=100) sets, including the number of true positives, false positives, and false negatives at the document level.](https://formative.jmir.org/api/download?filename=ba6f367047df85b010be5c898a4393b1.docx&alt_name=49721-776440-1-SP.docx)

|  | **TP** | **FP** | **FN** | **Precision (%)** | **Recall (%)** | **F1-Score (%)** |
| --- | --- | --- | --- | --- | --- | --- |
| **Training set** | 247 | 12 | 22 | 95.3 | 91.8 | 93.5 |
| **Development** | 237 | 23 | 14 | 91.1 | 94.4 | 92.7 |
| **Evaluation set** | 235 | 20 | 32 | 92.1 | 88.0 | 90.0 |
