# Supplementary material for: Automatic Extraction of Research Themes in Epidemiological Criminology From PubMed Abstracts From 1946 to 2020: Text Mining Study
Source: JMIR Form Res. 2023 Sep 22;7:e49721. doi: 10.2196/49721 (PMC10559193; doi:10.2196/49721)
Supplement: Multimedia Appendix 3 [file formative_v7i1e49721_app3.docx]

**Multimedia Appendix 3**

[The most common research priorities in 11,814 PubMed epidemiological criminology articles from 1946 to 2020.](https://formative.jmir.org/api/download?filename=50e4641f3d60cb592b54a723edd636dc.docx&alt_name=49721-776443-1-SP.docx)

| **PubMed theme** | **Number of PubMed articles** | **% All PubMed articles (n=11,814)** | **% PubMed articles related to Australia (N=679)** |
| --- | --- | --- | --- |
| Infectious diseases and infections | 2,949 | 24.9 | 23.3 |
| Mental health | 2,840 | 24.0 | 27.2 |
| *Other*^#^* | 2,552 | 21.6 | 20.2 |
| Alcohol and other drug use | 2,433 | 20.5 | 25.8 |
| *Biomedical related** | 1,388 | 11.7 | 11.8 |
| *Offence related** | 1,177 | 9.9 | 11.8 |
| Social determinants of health | 1,147 | 9.7 | 8.1 |
| *Behaviour** | 938 | 7.9 | 5.6 |
| Health care service | 911 | 7.7 | 6.0 |
| *Juveniles** | 502 | 4.2 | 1.5 |
| *Justice system** | 259 | 2.1 | 3.5 |
| Education | 172 | 1.4 | 0.6 |
| Women’s and maternal health | 13 | 0.1 | 0.1 |
| Nutrition | 1 | 0.0 | - |

^Non-italic: PubMed theme identified as stakeholder research priority.^

^* PubMed theme not identified as stakeholder research priority.^

^# Includes^ ^age, sex, gender, job satisfaction.^
