# Supplementary material for: Automatic Extraction of Research Themes in Epidemiological Criminology From PubMed Abstracts From 1946 to 2020: Text Mining Study
Source: JMIR Form Res. 2023 Sep 22;7:e49721. doi: 10.2196/49721 (PMC10559193; doi:10.2196/49721)
Supplement: Multimedia Appendix 4 [file formative_v7i1e49721_app4.docx]

**Multimedia Appendix 4**

[Distribution of PubMed articles (n=11,814) according to the categorized theme and income status of the country of research origin for the period from 1946 to 2020.](https://formative.jmir.org/api/download?filename=360175a644098b504d81d859a526b105.docx&alt_name=49721-776445-1-SP.docx)

|  | **High income (n=10,0083; 85.3%)** | **Upper middle income (n=740; 6.3%)** | **Lower middle income (n=435; 3.7%)** | **Low income (n=91; 0.8%)** |
| --- | --- | --- | --- | --- |
| **Infectious diseases and infections** | 2,270 | 291 | 186 | 47 |
| **Mental health** | 2,590 | 121 | 41 | 16 |
| **Other** | 2,217 | 145 | 94 | 20 |
| **Alcohol and other drug use** | 2,182 | 121 | 50 | 4 |
| **Biomedical related** | 1,155 | 90 | 85 | 8 |
| **Offence related** | 1,100 | 36 | 11 | 3 |
| **Social determinants of health** | 1,047 | 63 | 12 | 3 |
| **Behaviour** | 851 | 41 | 15 | 1 |
| **Health care service** | 794 | 41 | 29 | 13 |
| **Juveniles** | 464 | 18 | 5 | 0 |
| **Justice system** | 217 | 12 | 13 | 2 |
| **Education** | 131 | 19 | 11 | 2 |
| **Women and reproductive health** | 12 | 1 | 0 | 0 |
| **Nutrition** | 1 | 0 | 0 | 0 |
